# Neuroinflammation as a Factor of Neurodegenerative Disease: Thalidomide Analogs as Treatments

**DOI:** 10.3389/fcell.2019.00313

**Published:** 2019-12-04

**Authors:** Yoo Jin Jung, David Tweedie, Michael T. Scerba, Nigel H. Greig

**Affiliations:** Drug Design & Development Section, Translational Gerontology Branch, Intramural Research Program, National Institute on Aging, National Institutes of Health, Baltimore, MD, United States

**Keywords:** neuroinflammation, tumor necrosis factor-α (TNF-α), neurodegeneration, thalidomide, lenalidomide, pomalidomide, apremilast, immunomodulatory imide drugs (IMiDs)

## Abstract

Neuroinflammation is initiated when glial cells, mainly microglia, are activated by threats to the neural environment, such as pathogen infiltration or neuronal injury. Although neuroinflammation serves to combat these threats and reinstate brain homeostasis, chronic inflammation can result in excessive cytokine production and cell death if the cause of inflammation remains. Overexpression of tumor necrosis factor-α (TNF-α), a proinflammatory cytokine with a central role in microglial activation, has been associated with neuronal excitotoxicity, synapse loss, and propagation of the inflammatory state. Thalidomide and its derivatives, termed immunomodulatory imide drugs (IMiDs), are a class of drugs that target the 3′-untranslated region (3′-UTR) of TNF-α mRNA, inhibiting TNF-α production. Due to their multi-potent effects, several IMiDs, including thalidomide, lenalidomide, and pomalidomide, have been repurposed as drug treatments for diseases such as multiple myeloma and psoriatic arthritis. Preclinical studies of currently marketed IMiDs, as well as novel IMiDs such as 3,6′-dithiothalidomide and adamantyl thalidomide derivatives, support the development of IMiDs as therapeutics for neurological disease. IMiDs have a competitive edge compared to similar anti-inflammatory drugs due to their blood-brain barrier permeability and high bioavailability, with the potential to alleviate symptoms of neurodegenerative disease and slow disease progression. In this review, we evaluate the role of neuroinflammation in neurodegenerative diseases, focusing specifically on the role of TNF-α in neuroinflammation, as well as appraise current research on the potential of IMiDs as treatments for neurological disorders.

## Introduction

Inflammation is initiated as the body’s immune cells activate inflammatory cascades to prevent tissue damage from injury or infiltrating antigens. When successful, the inflammatory response eradicates pathogens, instigates the processes of wound healing and angiogenesis, and then subsides. Contrary to this protective homeostatic action, inflammation has been observed in a multitude of diseases, and its impact on neurological disorders has been proposed as a leading cause of disease progression in recent years. Within the CNS, microglia, known as ‘the brain’s immune cells,’ interact with astrocytes and neurons by assuming phagocytic phenotypes and releasing inflammatory cytokines. This can cause neurodegeneration, phagocytosis of synapses, diminished neural function, microglial activation, inflammatory cytokine release, and further microglial activation until threat to the neural environment abates. Activation of astrocytes, termed astrogliosis, also occurs as part of the inflammatory process. When acute, this neuroinflammatory response is necessary and even beneficial to the neural environment in eliminating pathogens or aiding brain repair. However, when extreme threats to the neural environment such as protein aggregates (i.e., lewy bodies, neurofibrillary tangles) accumulate in the brain and protractedly sustain inflammation, continuous gliosis and apoptosis can occur as a result of unregulated inflammatory cytokine release. Continuity of this activated state results in chronic inflammation, which is implicated in virtually all neurological disorders, including AD, PD, and ALS.

The majority of neurodegenerative diseases affect the aging population, which is growing at an exponential rate due to medical advancements that have prolonged lifespan (He et al., 2015). For example, AD is projected to affect an estimated 13.8 million people over the age of 65 in the U.S. alone during the next few decades (Alzheimer’s Association, 2019), while the number of PD patients is expected to rise to 1.24 million people in the next decade (Marras et al., 2018). Despite considerable effort and resources, drug developments to combat these neurodegenerative disorders have resulted in a mere handful of drugs that mitigate symptoms rather than slow disease progression (Mehta et al., 2017). There are numerous reasons why clinical trials have thus far largely failed, including the recruitment of patients too far into disease progression. However, the greatest reason for failure may be the selection of the disease target, exemplified by the predominant focus on inhibiting amyloid or tau aggregation for reversing AD and reducing α-synuclein to treat PD. There is a great need to expand target mechanisms to encompass prevention of chronic neuroinflammation, which has been elucidated as a key factor driving neurological disease progression through multiple cascades that include processes impacting the classical targets of amyloid, tau and α-synuclein.

## Mechanism and Signaling Events of Neuroinflammation in CNS Neurodegenerative Diseases

In response to deleterious stimuli such as injury or dysregulation in the CNS, proinflammatory cytokines and in particular, TNF-α, are released by brain endothelial cells, neurons, infiltrating immune cells, and resident glial cells (Sébire et al., 1993; Gadient and Otten, 1994; Ringheim et al., 1995; Reyes et al., 1999). This leads to activation of transcription factors by MAPKs, namely ERK, JNK, and p38, causing the upregulation of oxidative stress inducers and TNF-α, and the activation of microglia, astrocytes, and the NF-κB pathway (von Bernhardi et al., 2015). The NF-κB pathway can be canonically or non-canonically activated. Canonical activation involves direct NF-κB activation by TNF-α or proinflammatory triggers, such as ROS, and leads to increased proinflammatory gene expression. Canonical activation is necessary for a fast and acute response to both peripheral and central threats and injuries. In contrast, non-canonical activation occurs in response to various stimuli such as lymphotoxin and is slower, with specific, long-term effects. Lymphotoxin is a proinflammatory cytokine that activates the NF-κB pathway, initiating changes in gene expression crucial for not only inflammation, but also for processes such as lymph node development and gastrointestinal immune homeostasis (Bauer et al., 2012). Although most NF-κB activation is canonical, non-canonical activation has been implicated in neural stem cell pluripotency and possibly differentiation (Yang et al., 2010; Zhang and Hu, 2012; Xiao et al., 2018).

Whereas an inflammatory response is fundamental to successfully fight off disease and reestablish homeostasis in response to injury, an acute inflammation can become chronic and exacerbate disease when inflammatory pathways are mis-regulated. Neuronal NF-κB activation generally promotes cell survival and can mediate glial cell activation, proliferation, and inflammation (Tansey and Goldberg, 2010). When the PI3K/AKT/mTOR pathway is activated, the NF-κB sequestering protein IKB is released and NF-κB proinflammatory target genes are expressed, further stimulating the inflammatory pathway (Lokensgard et al., 2000; Kaltschmidt et al., 2005; Mémet, 2006). Proinflammatory cytokines can induce the expression of iNOS or COX-2 and lead to ROS production (Hirsch and Hunot, 2009). ROS cause oxidative damage that, in turn, can cause DNA damage, shortening of telomeres, increased apoptosis, and aging. A rise in ROS can lead to increased autophagy, which is consistent with the increased Aβ autophagy and dysfunctional mitochondria observed in AD patients (Cui et al., 2012). Oxidative damage results in an increase in ROS, which leads to yet further oxidative damage and feeds this self-propagating cycle (Cui et al., 2012). ROS may also trigger protein misfolding, potentially leading to protein aggregation, which is a classical hallmark of neurodegenerative diseases such as AD and PD (Narhi et al., 1999; Virmani et al., 2013). When cytokines, PAMPs, and DAMPs stimulate receptors such as TLR to canonically activate the NF-κB pathway, genes expressing proinflammatory cytokines are triggered, activating the nervous system’s immune response (Jana et al., 2008; Baker et al., 2011; Clark and Vissel, 2018). This leads to inflammation of the CNS, which is maintained until the brain environment reaches conditions to trigger deactivation of the immune response.

The presence of proinflammatory cytokines can also cause a shift or polarization of resting microglia to adopt their activated states. Microglial activation is routinely divided into two main phenotypes, M1 and M2, albeit that microglia can lie anywhere within the broad spectrum between these states (Town et al., 2005; Mosser and Edwards, 2008). M1 macrophages are activated by IFN-γ, IL-6, ROS, iNOS, and TNF-α in what is referred to as classical activation. Classical activation results in proinflammatory cytokine release, lymphocyte recruitment, and a strengthened immune response. M2 macrophage activation, also referred to as alternative activation, has neurotrophic and anti-inflammatory effects and leads to tissue repair. Alternative activation occurs as a result of stimulation by IL-4/10/13, and TGF-β. The M1:M2 phenotypic ratio is greater in times of injury, and higher ratios have been associated with chronic inflammation (Colton et al., 2006; Baker et al., 2011; Tang and Le, 2016).

Whereas mild levels of TNF-α and oxidative species may be neuroprotective and anti-apoptotic, excessive or prolonged neuroinflammation can be detrimental to brain health (Arends et al., 2000; Lee et al., 2010; Wenzel and Klegeris, 2018). In this regard, inflammation may be either protective or harmful depending on factors that include the activation cause, intensity, target, duration, transcriptional changes, and resulting intercellular activities (Scherbel et al., 1999; Gabbita et al., 2012). Additional elements such as increased blood-brain barrier (BBB) permeability along with CNS recruitment of peripheral lymphocytes can exacerbate chronic inflammation and cause a prolonged autoimmune-like response to injury (Hirsch and Hunot, 2009; Van Eldik et al., 2016). Continuous dysregulation of cytokine production can result in neurotoxicity and disrupt neural function (Hanisch, 2002).

As excessive neuroinflammation can impair neural function and induce neurotoxicity, regulating cytokines and restoring the brain microenvironment to pre-inflammatory conditions is crucial for reestablishing brain health following neural injury. Lowering and inhibiting proinflammatory cytokines, enhancing the activity of anti-inflammatory cytokines, effective phagocytic cell clearance, and selective inactivation of NF-κB can constrain inflammation and limit neuronal dysfunction and demise in neurodegenerative models (Baker et al., 2011; Song and Suk, 2017; Kabba et al., 2018).

## The Role of Inflammation and TNF-α in Neurodegenerative Disease (Figure 1)

**FIGURE 1:**
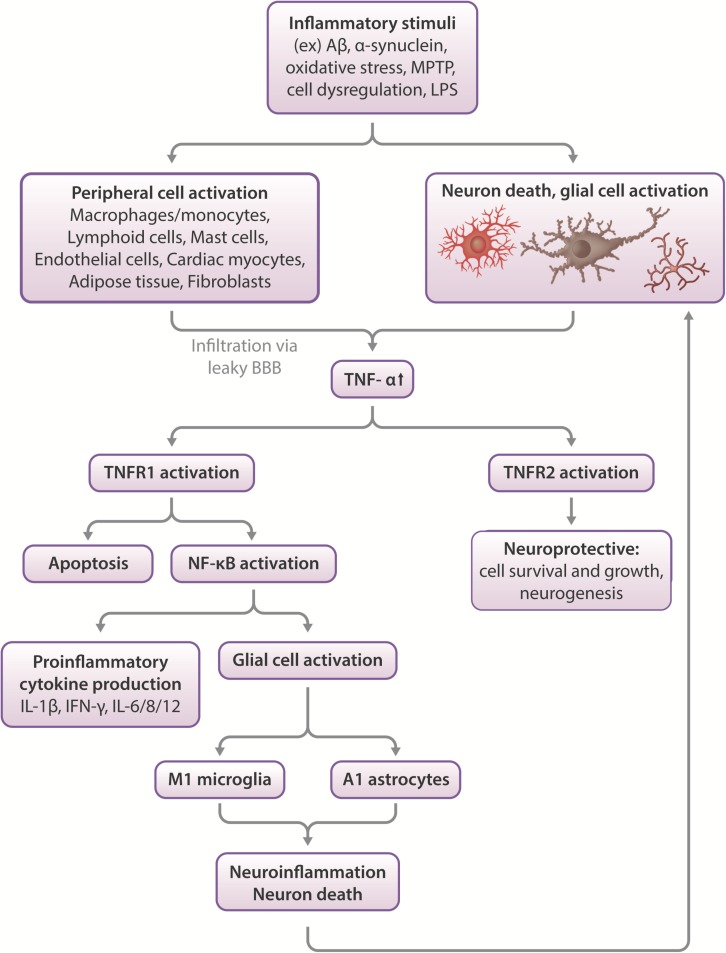
Effects of TNF-α in the CNS. Inflammatory stimuli such as Aβ, α-synuclein or oxidative stress can cause neuronal death and glial cell activation, which leads to the release of inflammatory cytokines such as TNF-α. In severe cases, peripheral macrophages can infiltrate the compromised blood-brain barrier to propagate an immune response in the CNS. TNF-α activates two types of receptors- TNFR1 and TNFR2. These function differentially, with TNFR1 promoting apoptosis and activating NF-κB, which leads to proinflammatory cytokine production and glial activation, which leads to neuroinflammation and neuronal death. More glial cell activation occurs in response to this pathway, feeding the positive feedback loop of increased neuroinflammation leading to increased cell death and vice versa. On the other hand, TNFR2 activation appears largely to be neuroprotective, stimulating neurogenesis and promoting cell survival and growth (Chen and Palmer, 2013; Longhi et al., 2013).

TNF-α is a proinflammatory cytokine produced by monocytes in the periphery and microglia, neurons, and astrocytes in the CNS (Clark et al., 2010). As a key player in inflammation, TNF-α acts as a major regulator of acute phase inflammation, initiating inflammatory cytokine signaling cascades (Parameswaran and Patial, 2010). Outside of the context of injury and inflammation, a lower level of constitutively expressed TNF-α is suggested to have physiological roles affecting fetal development, cardiovascular health, and various components of the immune system, including hematopoietic cell regeneration, affecting development and homeostasis of peripheral systems (Haider and Knöfler, 2009; Mizrahi and Askenasy, 2014; Urschel and Cicha, 2015). In the neural environment, constitutive physiological levels of TNF-α regulate synaptic plasticity, both in the context of injury (Liu Y. et al., 2017; Rizzo et al., 2018), development, and homeostasis (Gilmore et al., 2004; Lewitus et al., 2016; Liu Y. et al., 2017). TNF-α modulates dendritic maturation, pruning, and synaptic connectivity to respond to alterations in sensory stimuli to maintain homeostatic plasticity (Kaneko et al., 2008; Lewitus et al., 2016; Yee et al., 2017). One key mechanism regulating this is the cell-surface expression of AMPA receptors, a type of glutamate receptor regulating the influx of Ca^2+^ and Na^+^ in action potential initiation. AMPA receptors, whose trafficking is regulated by TNF-α, are key players in excitatory neurotransmission and long-term potentiation (LTP). Specifically, glial TNF-α has been shown to improve synaptic efficacy by increasing the cell-surface expression of AMPA receptors. Conversely, blocking TNF-α-mediated signaling has the opposite action. Notably, whereas TNF-α can adjust AMPA receptor cell surface expression, LTP and LTD are unaffected by TNF-α, but are regulated by physiological levels of the proinflammatory cytokine IL-1β. In contrast, TNF-α continuously adjusts synaptic scaling to keep the postsynaptic balance of excitatory synapses around a firing rate set point for optimal efficiency. In this manner, synaptic scaling provides a homeostatic form of synaptic plasticity, elicited to globally diminish (downscaling) or elevate (upscaling) the excitatory drive during chronic inactivity or hyperactivity, respectively, to maintain optimal neuronal performance (Liu Y. et al., 2017; Rizzo et al., 2018).

In response to injury, TNF-α functions to restore brain homeostasis during acute inflammation, acting as a defensive guard to protect against CNS injury, infection, neurodegeneration, and neurotoxicity. As with excessive production of other proinflammatory cytokines, unregulated TNF-α expression contributes to chronic inflammation, glutamatergic toxicity, synaptic loss, and excessive gliosis. Chronic TNF-α expression is observed in MS, ALS, PD, ischemia, HIV-associated dementia, and AD (Decourt et al., 2016; Hu et al., 2019).

All neurodegenerative diseases possess a neuroinflammatory component (Lee et al., 2010). A rise in brain TNF-α, for example, has been described across neurodegenerative disorders and may play a role in not only disease pathology and progression, but also the advancement of some of these diseases into others. An injury to the CNS in the event of a TBI can, for instance, lead to chronic inflammation that fuels Aβ misfolding and an increased risk for AD, as observed in post-mortem studies of TBI patients (Roberts et al., 1994; Ikonomovic et al., 2004; DeKosky et al., 2007). As a prolonged or chronic inflammatory response can induce an environment that is susceptible to dysfunction, TNF-α may be an ideal drug target as a treatment strategy to mitigate neurodegenerative diseases (Ramos-Cejudo et al., 2018).

### Traumatic Brain Injury

In response to TBI, cell death in the meninges, blood vasculature, and parenchyma is observed in the immediate impact area (DeWitt and Prough, 2003; Roth et al., 2014). Following primary injury, secondary injury is propagated by the resulting increase in ROS (Hall et al., 1994), glutamatergic toxicity (Bullock et al., 1995), and inflammation (Shi et al., 2019). Weakening of the BBB in response to injury can lead to CNS infiltration of peripheral monocytes and subsequent transformation into macrophages, as described in a study of controlled cortical impact (CCI) murine models (Hsieh et al., 2013).

Inflammation allows the CNS to respond to challenges such as TBI, and in ideal situations, reinstate neural function. To elucidate the role of TNF-α in TBI, TNF-α was knocked out in mice, after which cognitive performance was measured. Results showed that in the acute post-injury phase, TNF-α (−/−) mice performed better, but later, these same animals functioned worse than wildtype mice, with the TNF-α (−/−) mice exhibiting no cognitive recovery and a greater cortical injury volume (Scherbel et al., 1999; Longhi et al., 2013). The TNF-α receptor (TNFR) has two different subtypes, TNFR1 and TNFR2, which produce different effects when activated. Activation of neuronal/microglial TNFR1 contributes to neuroinflammation and degeneration, while activation of TNFR2 largely protects against the consequences of CNS injury, such as toxic accumulation of ROS/Ca^2+^, via the NF-κB pathway (Barger et al., 1995; Fontaine et al., 2002; Chen and Palmer, 2013; Longhi et al., 2013). TNFR1/2 KO mice exhibit increased vulnerability to ischemia and seizure-induced neuron damage (Bruce et al., 1996), indicating the need to selectively activate TNFR2 and deactivate TNFR1 at specific timepoints following injury to promote recovery.

### Alzheimer’s Disease

Alzheimer’s disease is an age-related neurodegenerative disease characterized by Aβ plaque and neurofibrillary tangle formation. Aβ aggregation is initiated in the frontal neocortex, including the orbitofrontal and transentorhinal cortices (Braak and Braak, 1991; Thal et al., 2002), and accumulates between neurons, resulting in synaptotoxicity (Dubnovitsky et al., 2013; Nieweg et al., 2015). As Aβ plaques can form more than a decade prior to cognitive symptom appearance in AD patients, numerous studies report tau pathology to correlate better with cognitive decline (Bejanin et al., 2017; Mielke et al., 2017). Similar to Aβ aggregation, tau tangles begin formation in the entorhinal cortex and manifest in areas of the brain controlling higher order cognitive processes such as memory formation and decision making (Pooler et al., 2013). Recent evidence suggests Aβ aggregates propagate tau tangle formation and the combined pathology of Aβ and tau aggregates lead to cognitive decline (Hanseeuw et al., 2019).

Multiple genetic and environmental factors contribute to AD risk. Some of these factors enhance neuroinflammation and hasten disease progression (Duyckaerts et al., 2009). APOE-ε4, a genetic risk factor for AD, intensifies brain atrophy. Rodents with the APOE-ε4 allele have higher proinflammatory cytokine release in response to LPS stimulation (Fernandez et al., 2019), indicating microglial hypersensitivity associated with the presence of APOE-ε4. Deactivation of another gene that aids in AD prevention, PIN1, leads to pTau accumulation and an increase in neuroinflammation (Clark and Vissel, 2018). Furthermore, activated glial cells have been widely found in post-mortem brains of AD patients (Heneka et al., 2015), indicating the presence of inflammation in late term AD brains.

Inflammation is also observed throughout AD progression and directly contributes to AD pathology. Early neuronal changes in AD pathology include increased production of prostaglandins, which stimulate inflammation at injury sites. Microglial activation is observed prior to cerebral neurodegeneration and astrocyte activation (Cagnin et al., 2001; Vehmas et al., 2003), and brain inflammation occurs early in preclinical AD patients (Monson et al., 2014). In middle to late stages of the disease, Aβ and pTau accumulate and astrocyte activation ensues (Vehmas et al., 2003). Neuroinflammation and cytokine production continue to increase as a result (Hoozemans et al., 2006). NF-κB has been shown to regulate the transcription of BACE1, an enzyme with an essential role in APP cleavage, due to the proximity of the BACE1 promoter to several NF-κB binding sites (Sambamurti et al., 2004). Upregulation of BACE1 leads to increased γ-secretase-mediated cleavage of APP, which leads to the generation and accumulation of Aβ (reviewed in Cole and Vassar, 2008), which in turn leads to increased inflammation, to yield a positive feedback loop of increased pathology (Combs et al., 2001; Kinney et al., 2018). There are also several signal transduction pathways linking cytokines such as TNF-α, IL-1, and IFN-γ with APP processing and subsequent Aβ plaque formation, as well as tau tangle formation (Griffin et al., 2006; Yamamoto et al., 2007; Shaftel et al., 2008). Due to the complex and broad impact that inflammatory processes can have in AD, theories such as the ‘damage signals hypothesis’ have arisen. This hypothesis theorizes that accumulation of cell stress from oxidative stress or neural injury results in long-term innate immune system activation/chronic neuroinflammation that instigates neurodegeneration, and ultimately leads to AD (Maccioni et al., 2009). It thereby provides a unifying mechanism to account for the diversity of risk factors that over long periods of time can bring different individuals into common pathways leading to AD.

To date, Aβ and pTau have been the most popular targets in AD drug development and research. However, emerging views of neural Aβ and pTau accumulation as disease hallmarks rather than the initial causes of AD, as well as the lack of success in AD drug development using these targets have led to a search for new drug targets. *In vivo* positron emission and single-photon emission computed tomography (PET and SPECT) scans of AD patients and AD transgenic mouse studies have pointed to neuroinflammation as a biomarker for disease progression and severity, allowing for the possibility of more accurate prediction of cognitive decline in preclinical or early AD patients (Hamelin et al., 2018; Focke et al., 2019). This suggests the need to look into factors of inflammation as potential therapeutic targets for AD. TNF-α, a key and initiating element in neuroinflammation, is known to activate various parts of the amyloid pathway, which underpins a key component of AD pathology. Hence targeting TNF-α, which appears to be both involved throughout both early and late stages of the cascades that trigger Aβ accumulation, may lead to a viable treatment for AD (Sriram and O’Callaghan, 2007; Clark et al., 2010; Clark and Vissel, 2018).

Recent research showing the positive effects of physical exercise, IL-6 supplementation, and anti-inflammatory medications to reduce TNF-α in AD models supports the premise that lowering TNF-α may mitigate or prevent AD pathology (Decourt et al., 2016). In addition, the increasing number and sophistication of ligands that permit time-dependent *in vivo* imaging of microglial and astrocyte activation, whether by PET or SPECT (for review see Edison et al., 2018), together with exosome technology to quantitatively follow inflammatory proteins enriched from brain derived exosomes available in the plasma (Pulliam et al., 2019) have the potential to serve for early diagnosis of AD, to monitor disease progression and to test the efficacy and the most effective time window for potential anti-inflammatory treatment strategies.

### Amyotrophic Lateral Sclerosis

Amyotrophic lateral sclerosis, a disease characterized by a loss of motor neurons in motor cortex, brainstem, and spinal cord, also demonstrates aspects of inflammation that may drive disease progression. Although the mechanisms through which ALS progresses remain to be more fully elucidated, mutations in ALS-associated genes such as C9orf72 or SOD1, which may activate microglia, increase risk of ALS (Brettschneider et al., 2012; Lall and Baloh, 2017). Activated microglia, astrocytes, and T cells have been found in all sites of motor neuron injury in ALS brains. ALS patients often generate immune responses to autoantigens, implying dysregulation of the immune system (Lall and Baloh, 2017). In addition, the over-activation of NF-κB and resulting inflammation leads to motor neuron degeneration in ALS disease models (Akizuki et al., 2013; Palotas et al., 2017).

Based on familial studies of ALS, C9orf72 mutations are the most common genetic cause of ALS, accounting for approximately 40% of familial ALS and 5–10% of sporadic ALS cases (DeJesus-Hernandez et al., 2011; Renton et al., 2011). C9orf72 is a protein thought to regulate endosomal trafficking (Farg et al., 2014), and its mutation was the first genetic link to frontotemporal dementia and ALS pathology. Some ALS cases have shown cognitive decline driven by TDP-43, a major source of ALS and FTD proteinopathy, and microglial activation in frontotemporal regions of the brain (Brettschneider et al., 2012). Rodent studies have shown links between reduced expression of C9orf72 and upregulation of TREM2, a protein expressed solely in microglia within the CNS and associated with increased phagocytosis of cell debris and pathogens (Lall and Baloh, 2017; Gratuze et al., 2018), leading to increased microglial activation and inflammation in the spinal cord (Fellner et al., 2017). Factors in CSF from ALS patients activate rat astroglial and microglial cultures, upregulating inflammatory cytokines, downregulating neuroprotective factors, and causing neurodegeneration in cocultures containing motor neurons (Mishra et al., 2016, 2017).

There is also substantial evidence behind the role for TNF-α in ALS. TNF- α is reported to be upregulated in the blood and CSF of ALS patients (Babu et al., 2008; Cereda et al., 2008), and TNFR1 and TNF-α have been found to be upregulated in the spinal cord of SOD1 mutant mice (Veglianese et al., 2006; Brambilla et al., 2016). According to genomic sequencing data, TNF-α is a major differentially expressed gene affecting protein interactions in ALS patients with C9orf72 mutations, making TNF-α a promising target for ALS treatment (Kotni et al., 2016). RNA sequencing data also points to TNF-α as a major regulator of upregulated inflammatory pathways in ALS patients’ spinal cords (Brohawn et al., 2016). Astrocyte and motor neuron cocultures from mice with mutations in genes linked to ALS, such as FUS or SOD1, demonstrate an increased release of TNF-α, leading to excitotoxicity-induced neurodegeneration. This degeneration can be blocked with TNF-α antibodies or synthesis inhibitors, such as thalidomide (Tortarolo et al., 2015; Kia et al., 2018).

### Parkinson’s Disease

Parkinson’s disease, an age-related neurodegenerative disorder, is distinguished by a progressive motor phenotype that encompasses tremors, rigidity, and bradykinesia. Neuropathologically, PD is characterized by the presence of Lewy bodies- intracellular proteinaceous inclusions that are chiefly comprised of insoluble α-synuclein aggregates. These aggregates induce deterioration of DA neurons in the *substantia nigra pars compacta* (SN) and fibers within the striatum (Polymeropoulos et al., 1997; Miller et al., 2004), as well as BBB dysfunction from increased production of inflammatory cytokines such as TNF-α and IL-6 (Dohgu et al., 2019). Neuroinflammation is implicated in PD as seen through results of post-mortem autopsies and molecular findings. Large numbers of activated microglia are universally observed in PD patient brains post-mortem, and PD brains and CSF have elevated levels of inflammatory factors such as TNF-α, IL-1β, IL-6, and IFN-γ. Furthermore, the densest population of microglia in the brain appears to lie in the midbrain with DA neurons, which are particularly sensitive to cytokines and oxidative stress. Astrocyte exposure to α-synuclein results in astrogliosis and sensitization of the inflammatory pathway, which ultimately leads to neuronal death in neuron and astrocyte cocultures (Chavarría et al., 2018; Hughes et al., 2019).

Neuroinflammation intensifies with disease progression (Mccoy et al., 2006; Hirsch and Hunot, 2009), and there is a multitude of research suggesting inflammation can trigger PD pathology. Some of this research revolves around associations of immune function-related genes [i.e., LRRK-2, HLA-DR, Nurr1 (Gardet et al., 2010; Hamza et al., 2010; Decressac et al., 2013)] and high levels of phosphorylated α-synuclein proteins with higher risk and incidence of PD. Other theories revolve around the possibility of α-synuclein aggregating in the olfactory bulb or gut lumen due to inflammatory processes and being spread to the striatum via the olfactory tract, vagus nerve, or spinal cord through a prion-like propagation (Hawkes et al., 2007; Greene, 2011). There is also evidence of greater α-synuclein aggregation in response to oxidative stress, which induces inflammation (Myöhänen et al., 2012; Scudamore and Ciossek, 2018). In this regard, PD patients may be more sensitive to chronic inflammation, as it may promote α-synuclein aggregation (Brundin et al., 2008; Hirsch and Hunot, 2009; Lema Tomé et al., 2013).

Several treatments focused to mitigate neuroinflammation have been shown to positively impact PD models. Early treatment with NSAIDs or other anti-inflammatory drugs has been shown to delay or prevent PD onset (Tansey and Goldberg, 2010). TNF-α inhibitors have been shown to block neurotoxin-induced nigral DA neuron loss by 50% (Mccoy et al., 2006) and attenuate reductions in striatal dopamine in MPTP rodent models (Boireau et al., 1997; Ferger et al., 2004), highlighting the potential use of these agents as treatments for PD.

### Multiple Sclerosis

Multiple sclerosis is a neurological demyelinating disorder in which immune cells from the periphery infiltrate boundaries of the CNS and attack the myelin sheath coating axons, leading to myelin, axonal, and neuronal degeneration (Shechter et al., 2013). The pathological basis of disease initiation remains an area of critical investigation, and it is unclear as to whether MS originates in the CNS, where degeneration of oligodendrocytes triggers activation and infiltration of peripheral immune cells, or if MS is initiated by autoimmune mechanisms within the periphery (Stys et al., 2012; Ghasemi et al., 2017).

Microglial activation takes part in the prevention and repair of MS in its early stages, but also acts to exacerbate disease by releasing inflammatory cytokines to increase peripheral immune cell recruitment and induce demyelination (Rawji and Yong, 2013). Of the inflammatory cytokines involved in MS progression, TNF-α has been at the forefront of MS pathology, with autopsy results showing elevated TNF-α levels in areas of active lesion formation (Hofman et al., 1989). An increase in TNF-α levels in the CSF of patients with chronic progressive MS, as compared to those with stable MS, has also been observed, indicating a link between heightened TNF-α levels and MS progression (Sharief and Hentges, 1991). Reductions in proinflammatory cytokines, including TNF-α, have been shown to decrease demyelination and immune cell infiltration into the CNS in models of experimental autoimmune encephalomyelitis (EAE), one of the primary models of MS in preclinical research (Nomura et al., 2011; Haghmorad et al., 2019).

Although TNF-α blockers have shown promising results in EAE models, clinical trials of TNF-α-targeting drugs have proven to be ineffective and even detrimental in MS patients (Van Oosten et al., 1996). There have even been reports of MS onset in TNF-α-blocker therapy for treatment of other diseases (Titelbaum et al., 2005; Al Saieg and Luzar, 2006; Pfueller et al., 2008; Bradshaw et al., 2016), pointing to the sensitivity of MS to levels of systemic TNF-α. These detrimental effects could be due to the non-selective inhibition of TNF-α (Williams et al., 2014; Steeland et al., 2017; Pegoretti et al., 2018; Sanadgol et al., 2018; Valentin-Torres et al., 2018; Fischer et al., 2019), pointing to the need for research that elucidates the subtype and time-dependent roles of TNF-α and inflammation in pathways relating to myelination.

## History and Mechanism of Thalidomide (Figure 2)

**FIGURE 2:**
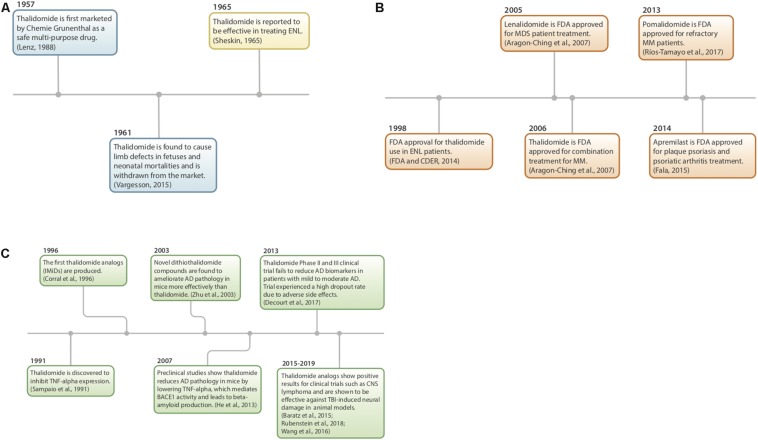
**(A–C)** History of thalidomide and IMiDs. **(A)** Thalidomide discovery, market withdrawal, and repurposing (Sheskin, 1965; Lenz, 1988; Vargesson, 2015). **(B)** FDA approval of IMiDs (Aragon-Ching et al., 2007; FDA and CDER, 2014; Fala, 2015; Ríos-Tamayo et al., 2017). **(C)** Preclinical studies using IMiDs (Sampaio et al., 1991; Corral et al., 1996; Zhu et al., 2003; Baratz et al., 2015; Wang et al., 2016b; Decourt et al., 2017; Liu T. et al., 2017; Rubenstein et al., 2018).

Thalidomide (α-phthalimidoglutarimide) was first marketed by Chemie-Grunenthal in 1957 under the drug name “Contergan.” It was advertised as a safe, multipotent drug capable of treating symptoms ranging from anxiety to hyperthyroidism (Lenz, 1988; Vargesson, 2015). It was prescribed to pregnant women in Europe as a well-tolerated morning sickness treatment. However, after 4 years and approximately 10,000 babies born with congenital limb deficiencies, thalidomide was taken off of the market due to its teratogenic effects (McBride, 1961; Vargesson, 2015).

Repurposing of the drug began more than 50 years ago as a serendipitous treatment for erythema nodosum leprosum (ENL), a skin disorder caused by leprosy (Sheskin, 1965). Since then, thalidomide has been discovered to have anti-angiogenic and anti-inflammatory properties, with the potential to treat a multitude of diseases, such as AIDS, certain cancers, and rheumatoid arthritis. Currently, it is being used to treat multiple myeloma, refractory anemia and Crohn’s disease (Wilhelm et al., 2006; Kurtin and List, 2009).

**FIGURE 3:**
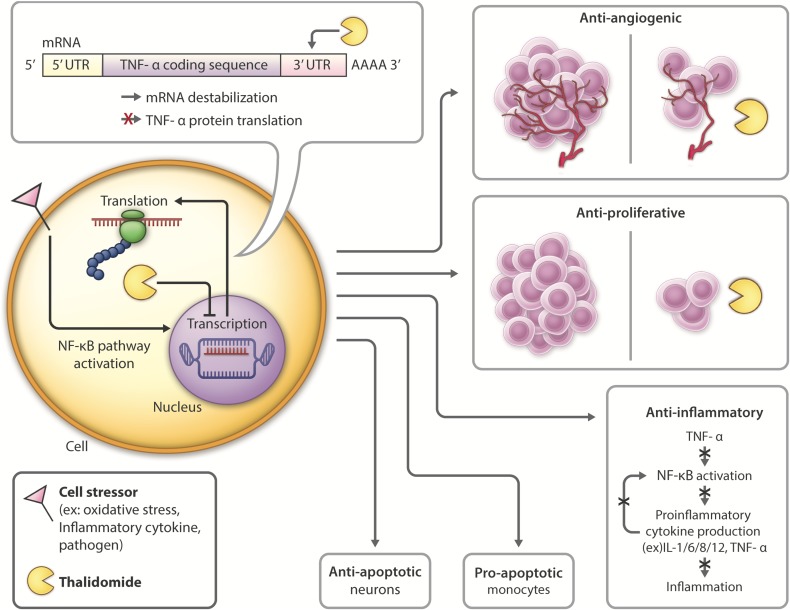
Thalidomide mechanism of action and pleiotropic effects: Following introduction of cell stressors such as inflammatory cytokines or pathogens, the NF-kB transcription factor is activated, leading to increased transcription and translation of TNF-α. Thalidomide binds to the 3′-UTR of TNF-α mRNA, leading to mRNA destabilization and inhibiting TNF-α cytokine production. Thalidomide has anti-angiogenic (D’Amato et al., 1994; Zeldis et al., 2011) and anti-proliferative (Arrieta et al., 2002; Zeldis et al., 2011; Mendy et al., 2012) properties, inhibiting tumor growth, and making it a promising candidate for cancer treatment. Thalidomide also possesses anti-inflammatory (Sampaio et al., 1991; Zeldis et al., 2011; Farfán et al., 2015) properties, which have the potential to be used in inhibiting inflammation contributing to neurological disease. Thalidomide has differential roles in the periphery versus the CNS, activating anti-apoptotic (Baratz et al., 2015; Farfán et al., 2015; Tsai et al., 2018) pathways in neurons and pro-apoptotic (Mitsiades et al., 2002; Gockel et al., 2014) in monocytes.

In 1991, thalidomide was discovered to inhibit TNF-α synthesis (Sampaio et al., 1991) and as a consequence, novel analogs, often referred to as IMiDs, have been synthesized and evaluated in the hope of enhancing drug potency and decreasing side effects. Thalidomide and analogs have been found to possess a broad range of actions, including anti-angiogenic, anti-proliferative, and anti-inflammatory effects (Zeldis et al., 2011; Figure 3).

In the periphery, thalidomide has anti-angiogenic and anti-proliferative properties (Arrieta et al., 2002), making it a potential drug for treatment of diseases such as cancer (D’Amato et al., 1994). Thalidomide was FDA approved to treat refractory multiple myeloma in 2013 due to its induction of apoptosis of myeloma cells and ability to inhibit bone marrow support to myeloma cells by impairing cell adhesion (Mitsiades et al., 2002; Vallet et al., 2008). Thalidomide also has analgesic properties shown in models of neuropathic pain, attributed to its ability to attenuate TNFR expression (Andrade et al., 2012).

Within the CNS, thalidomide has been shown to reduce the generation and secretion of inflammatory cytokines such as IL-1, IL-6, IL-8, IL-12, GM-CSF and TNF-α (Vallet et al., 2008; Majumder et al., 2012). The drug downregulates NF-κB in activated B cells (Keifer et al., 2001; Yagyu et al., 2005; Paul et al., 2006; Li et al., 2009) and degrades TNF-α mRNA by altering it at its 3′-UTR (untranslated region) to destabilize the cytokine post-transcriptionally, ultimately shortening its half-life (Moreira, 1993; Zhu et al., 2003; Figure 3). With lower TNF-α levels, proinflammatory cytokine production is inhibited, and neuroinflammation is reduced. Thalidomide has also been shown to have anti-oxidative and anti-apoptotic effects in stroke and TBI models (Farfán et al., 2015; Tsai et al., 2018).

**FIGURE 4:**
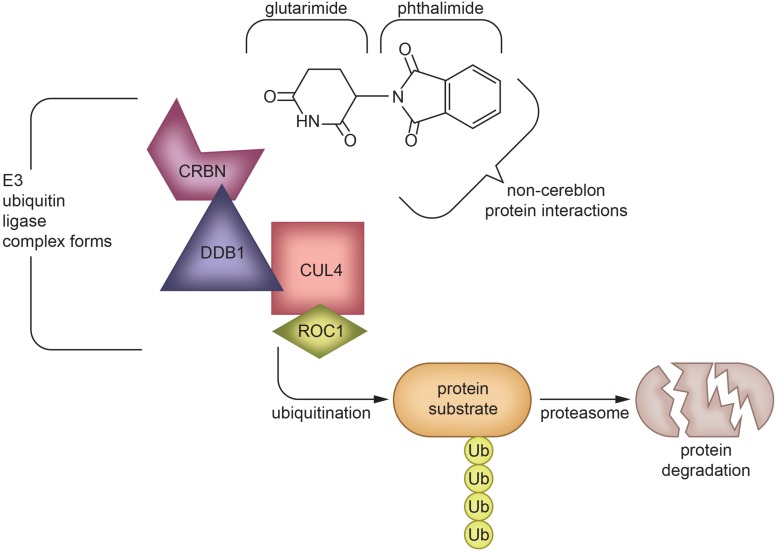
Protein degradation mechanism of Thalidomide via cereblon-binding: Thalidomide is composed of glutarimide and phthalimide moieties, which interact differentially with various targets. The glutarimide ring binds cereblon to create an E3 ubiquitin ligase complex (Mendy et al., 2012), while phthalimide targets non-cereblon proteins (Noguchi et al., 2004). The formation of an E3 ubiquitin ligase complex leads to protein degradation (Winter et al., 2015; An and Fu, 2018), which underlies the ability of IMiDs to treat multiple myeloma (Krönke et al., 2014) and potentially other diseases in which protein aggregation or malfunction play a role. The exact mechanism of TNF-α inhibition by IMiDs is yet to be elucidated, but is likely to be a combination of effects of both glutarimide and phthalimide moieties (Schett et al., 2010; Millrine and Kishimoto, 2017; Chelucci et al., 2019). CRBN, Cereblon; DDB1, DNA damage-binding protein 1; CUL4, Cullin-4; ROC1, Regulator of Cullins-1.

Although thalidomide derivatives have varying properties and potencies, they all appear to lower TNF-α. A breakthrough in understanding potential mechanisms underpinning thalidomide and related IMiDs is that some appear to bind to cereblon, a critical component of an E3 ubiquitin ligase complex with an intermediary role in immunomodulation (Mendy et al., 2012; Figure 4). In the context of multiple myeloma, thalidomide analogs cause cereblon to interact with associated factors to form a ligase that breaks down Ikaros (IKZF1) and Aiolos (IKZF3), two members of the Ikaros family of zinc finger transcription factors important in B-cell development (Krönke et al., 2014). The protein degradation abilities of thalidomide via its interactions with cereblon have been an emerging topic of interest in the field of drug development (Winter et al., 2015; An and Fu, 2018). However, the implications and detailed mechanism of cereblon interactions with thalidomide’s anti-inflammatory response remain unclear (Millrine and Kishimoto, 2017). It is quite possible that IMiDs have both cereblon-dependent and independent mechanisms that impact TNF-α and its multiple associated proteins in neuroinflammation, as not all IMiDs with TNF-α inhibiting properties have cereblon binding potential (Min et al., 2016; Millrine and Kishimoto, 2017). It is clear, however, that cereblon and IKZF1/3 are expressed within the brain and are associated with learning and memory, with mutations in cereblon posing a risk for intellectual disabilities (Higgins et al., 2004; Chang and Keith Stewart, 2011). Interestingly, in an integrated multi-cohort transcriptional meta-analysis of neurodegenerative diseases in which genes associated with M1-polarized macrophages/microglia and reactive astrocytes were strongly enriched, IKZFI was identified across all of the neurodegenerative disorders evaluated (Li et al., 2014). Thalidomide has also shown anticonvulsant actions in seizure models of mice, possibly through the inhibition of iNOS (Payandemehr et al., 2014), which, likewise, has a role in inflammation (Mansouri et al., 2018).

## Clinical Trials of Thalidomide and Its Analogs in Neurodegenerative Disease

Thalidomide and its analogs have and are continuing to be evaluated in clinical trials that have led to the repurposing of IMiDs for diseases such as multiple myeloma. Thus far, the majority of research and clinical trials performed on novel thalidomide derivatives have related to peripheral diseases. The few trials that have had CNS disease targets (Table 1) have focused on the anti-angiogenic properties of IMiDs. For instance, NCT02415153 is a phase I trial in which the tolerability and best dose of pomalidomide in treating younger patients with persistent tumors of the brain or spine are being studied. NCT01553149 is a phase II trial that compares the effects of low and high doses of lenalidomide in treating younger patients with juvenile pilocytic astrocytomas or recurrent, refractory, or progressive optic nerve pathway gliomas.

**TABLE 1:** Clinical trials of Thalidomide and its FDA approved analogs relating to neurological disorders.

**Candidate**	**Condition**	**ClinicalTrials.gov Identifier(s)**	**Clinical phase**	**Results**
Thalidomide	Amyotrophic Lateral Sclerosis (ALS)	NCT00231140, NCT00140452	Pilot, Phase II	Study was terminated due to bradycardia occurrence as a common adverse effect (Meyer et al., 2008); no significant improvement in function or cytokine profile following 9 months of treatment (Stommel et al., 2009).
Thalidomide	Arachnoiditis	NCT00284505	Phase II	Unavailable
Thalidomide	Epilepsy	NCT01061866	Phase I, II	The mean number of seizures in the patients tested decreased from 27 ± 4 to 7 ± 1 seizures per month, showing a high therapeutic potential for thalidomide on refractory seizures (Palencia et al., 2010).
Thalidomide	CNS tumor and metastases	NCT00049361, NCT00527657, NCT00039468, NCT00179881, NCT00098865, NCT00006358, NCT00079092, NCT00014443, NCT00112502, NCT00047281, NCT00047294	Phase I, II, III	No beneficial effect of thalidomide on CNS metastases was observed. In a trial with thalidomide treatment combined with radiation therapy (NCT00049361), nearly half of the thalidomide treatment group discontinued treatment due to side effects (Knisely et al., 2008). When given in conjunction with radiation therapy and temozolomide, efficacy was low (Atkins et al., 2008; Penas-Prado et al., 2015) (NCT00112502). Some trials are ongoing (NCT00179881) or have no reported results.
Thalidomide	Adrenoleuko- dystrophy	NCT00004450	Orphan study	Immunosuppressive drugs combinations had no beneficial effect on patients (Berger and Gärtner, 2006).
Thalidomide	Alzheimer’s Disease	NCT01094340	Phase II, III	Thalidomide was poorly tolerated by trial participants, preventing patients from reaching the target dose of thalidomide and causing patients to drop out of the study prematurely (Decourt et al., 2017).
Lenalidomide	CNS tumor	NCT00036894, NCT03050450, NCT01553149, NCT00100880, NCT0370316, NCT03558750	Phase I, II	Lenalidomide proved to be well tolerated in CNS tumor patients (Fine et al., 2007) (NCT00036894), and was found to have antitumor activity in pediatric CNS tumor patients (Warren et al., 2010) (NCT00100880). Some trials are ongoing (NCT01553149, NCT03703167) or have no reported results.
Lenalidomide	POEMS Syndrome	NCT00971685, NCT01816620, NCT02193698, NCT02921893	Phase II	Lenalidomide treatment in conjunction with dexamethasone significantly relieved symptoms of POEMS, such as extravascular volume overload, organomegaly, and pulmonary hypertension (Li et al., 2018) (NCT01816620). NCT02921893 is ongoing, but preliminary data suggests ixazomib, dexamethasone, and lenalidomide combination therapy is effective at treating POEMS syndrome (Dispenzieri et al., 2019).
Lenalidomide	Neuropathy	NCT00665652	Phase II	Trial was terminated due to unexpected paraproteinemia side effect (Connolly et al., 2010).
Pomalidomide	CNS tumor	NCT02415153, NCT03257631, NCT03798314	Phase I, II	Pomalidomide treatment of children with CNS tumors failed to meet clinical significance (Fangusaro et al., 2019). NCT03798314 is ongoing.

However, these drugs have yet to be evaluated systematically in subjects of neurodegenerative disease. Thus far, thalidomide has been tested in ALS patients in 2 studies. One study (NCT00231140) was terminated prematurely due to safety concerns regarding a common sinus bradycardia effect in the patients (Meyer et al., 2008). Another study (NCT00140452) observed primarily adverse effects and no improvement in treated subjects (Stommel et al., 2009). Thalidomide has also been tested in AD patients, but no differences in BACE1, a genetic measure of AD severity, were observed and 14 out of 25 patients dropped out of the trial due to adverse drug effects (Decourt et al., 2017), preventing the trial from administering the necessary dose to see an efficacious response (NCT01094340). Although thalidomide trials of neurological diseases have been unsuccessful to date, forthcoming trials should be focused on more potent new generation IMiDs in treating neurological diseases as, clearly, improvements over thalidomide need to be made in new analogs before they can be used to effectively treat neuroinflammation. Drug dose adjustment to mitigate toxicity and enhance potency via an increased understanding of drug-to-target attraction and bioavailability should be made for future clinical studies. As an example of the latter, lenalidomide distribution into brain is surprisingly low, in contrast to thalidomide (Rozewski et al., 2012). Whereas the structure and lipophilicity of lenalidomide and thalidomide are similar, a recent study suggests that the former is a substrate of the efflux transporter, *P*-glycoprotein (Hofmeister et al., 2011), which actively removes select compounds from the brain as part of the BBB (Erickson et al., 2012). Future research focused on separating the anti-inflammatory from the primary adverse effects of IMiDs could potentially lead to repurposing of thalidomide analogs as treatments for neurodegenerative diseases such as AD, PD, ALS, or TBI.

## Advantages of Thalidomide Derivatives Over Other Drugs

Thalidomide analogs such as lenalidomide and pomalidomide are the lead IMiDs being tested in clinical trials (Bartlett et al., 2004a). One advantage of thalidomide is its high bioavailability (90% after oral dosing) and distribution to the brain (brain/plasma ratio 0.89 in rodent studies (Huang et al., 2005), CSF/plasma ratio 0.42 in non-human primates (Muscal et al., 2012). As noted, a key mechanism by which thalidomide inhibits TNF-α production is post-transcriptionally via mRNA degradation (Moreira, 1993; Rowland et al., 1999). TNF-α levels, like other proteins, are regulated both transcriptionally and post-transcriptionally. However, under healthy control conditions the translation of TNF-α mRNA into protein is highly repressed via RNA-binding proteins and miRNAs that interact with AREs within the 3′-UTR of TNF-α (Mazumder et al., 2010). This translational control provides a tactical benefit for cells, such as microglia, that generate and release proteins that have a physiological role (at routine low levels) and an immune role (at high concentrations), allowing a rapid change in roles through the use of pre-existing mRNAs to sidestep the lengthy nuclear control mechanisms (i.e., transcription, splicing, and transport). Simultaneously, it affords the equally rapid reversibility through modifications (e.g., reversible phosphorylation) of the regulatory intermediates. Collectively, this permits rapid activation or cessation of synthesis of a specific protein required for inflammation. Hence, in response to LPS or a similar challenge, a 3 to 4 log (1000 to 10,000-fold) increase in TNF-α protein secretion can be swiftly delivered by removal of the translational blockade and concurrent activation of transcription. A group of RNA-binding proteins are recruited to the ARE within the 3′-UTR of TNF-α that, collectively, stringently regulate translation. These include the RNA stabilizing protein HuR, heterogeneous nuclear ribonucleoprotein (hnRNP)-A1, T cell intracellular Ag (TIA)-1, TIA-1–related protein, AUF1 and tristetraprolin (TTP). For example, hnRNP-A1 binding to the ARE element represses TNF-α mRNA basal translation, and subsequent phosphorylation of hnRNP-A1 by Mnk, a kinase downstream of p38 MAPK, lowers its affinity for the ARE and, thereby, restores TNF-α translation (Mazumder et al., 2010). Likewise, TTP and AUF1 both destabilize TNF-α mRNA and thus reduce its translation into protein (Zhang et al., 2002; Lai et al., 2015). Both these RNA-binding proteins appear to be regulated by p38 MAPK, and p38 MAPK has been shown to be inhibited by thalidomide (Noman et al., 2009) – providing one of likely several mechanisms via which thalidomide and similar IMiDs regulate TNF-α levels. As TNF-α expression has been shown to be upregulated in models of neurological disease (Colton et al., 2006; Brohawn et al., 2016; Lindenau et al., 2017), supporting the need to target TNF-α to decrease neuroinflammation accompanying neurodegeneration, the high brain penetrance of thalidomide and analogs together with an ability to repress TNF-α at the level of its synthesis, provides a promising approach to mitigate neuroinflammation.

Support of TNF-α as a drug target for neurodegenerative disorders is provided by etanercept (Enbrel^®^) and infliximab (Remicade^®^). These TNF-α specific monoclonal antibodies and recombinant fusion proteins are widely used TNF-α modulators that have proven to be highly effective against a broad number of inflammatory-mediated diseases, including rheumatoid arthritis, Crohn’s disease, ulcerative colitis, psoriatic arthritis, plaque psoriasis and ankylosing spondylitis (for review see Sedger and McDermott, 2014; Monaco et al., 2015), and have been applied to neurological disorders. By acting as a false target and binding to soluble and membrane-bound TNF-α, these biologicals prevent TNF-α from interacting with its receptors, thereby precluding ligand triggered TNFR signaling. The application of the anti-TNF-α antibody chimeric monoclonal antibody approach to neurodegenerative disorders was pioneered by Tobinick (2010, 2018). In a 6-month open AD trial, etanercept administration demonstrated significant improvements in dementia patients (Tobinick et al., 2006), whereas infliximab has been reported to improve cognition in an AD case study (Shi et al., 2011), providing positive preliminary data that warrants support to follow up in a double blind study. Notably for systemic disorders like rheumatoid arthritis, both etanercept and infliximab require intravenous or subcutaneous injection for routine administration as they are biologicals that possess a low bioavailability and are subject to degradation if taken orally. As protein therapeutics, they have strictly limited BBB penetration, and are hence best administered by perispinal injection paired with Trendelenburg positioning- a head-down tilt placing to attempt to facilitate drug delivery into the CSF following their distribution into the cerebrospinal venous system (Tweedie et al., 2007; Tobinick, 2010, 2018; Sumbria et al., 2017; Clark and Vissel, 2018).

In contrast and as noted, thalidomide, is orally active, has a high bioavailability and readily permeates the BBB, allowing for its easy and less invasive administration. Studies comparing etanercept, infliximab, and thalidomide treatments are rare, but a preclinical one exists in a STZ-induced dementia rat model that demonstrated the efficacy of all three treatments to mitigate neurodegeneration. Thalidomide was favored as the treatment group by the studies’ authors as it demonstrated a trend for lowest neuritic plaque formation (Kübra Elçioğlu et al., 2015).

A promising feature of the above approaches is their pleiotropic nature, as TNF-α is tied into multiple signaling pathways that impact key mediators on inflammation. Unfortunately, TNF-α lowering agents are not a magic bullet for all inflammatory disorders. Adverse events can be severe in some patients. For anti-TNF-α antibodies, infections can develop, up to 20% of subjects do not initially respond to therapy, and immunogenicity can occur that leads to a lack of response in up to 46% of patients over 12 months of treatment (Ben-Horin et al., 2014). In contrast, IMiDs are known to be teratogenic and therefore cannot be prescribed to women who are pregnant or who may become pregnant during therapy. Thalidomide is associated with neutropenia, leukopenia and lymphopenia at high doses as well as with peripheral neuropathy and somnolence. Nevertheless, the repurposing or repositioning of a drug, if efficacious and tolerable, can be beneficial as it can lead to a rapid disease treatment with limited resources and/or a proof of mechanism study that, if positive, can then open up a new and focused drug development. The cost of clinical trials to demonstrate safety and efficacy of an original drug can take up to 15 years and $2.6 billion (DiMasi et al., 2016). In comparison, repurposing an existing drug provides an accelerated route in light of its known clinical safety and pharmacokinetic data – resulting in substantially lower costs [as low as $8.4 million and 3–12 years (Agrawal, 2015; Hernandez et al., 2017)] and a lower rate of attrition. As a pertinent example, thalidomide was successfully repurposed by Celgene for the treatment of a broad number of systemic inflammatory disorders and cancers. Thus far, however, the preclinical promise of thalidomide in animal models of neurodegeneration has not translated into clinical efficacy (Meyer et al., 2008; Stommel et al., 2009; Decourt et al., 2017) as sufficient CNS TNF-α and anti-inflammatory action appears unachievable in the absence of dose-limiting adverse effects. This, amongst other factors, provides a basis of evaluating both existing and new analogs that may pair higher potency with greater tolerability.

## Current Research Surrounding Thalidomide Analogs

Thalidomide has been modified to form several analogs, some of which have been widely tested for efficacy in treating various cancerous and immunological diseases. These new analogs have been generated to provide more TNF-α inhibition, a broader range of activities and, in some cases, a high BBB penetrance (Corral et al., 1996; Tweedie et al., 2007).

Not all thalidomide analogs have the same properties; for instance, analogs such as apremilast are PDE inhibitors, while others are not. While non-PDE inhibiting analogs stimulate T cells, IL-2, and IFN-γ production, PDE inhibiting analogs have little effect on peripheral T cells (Corral et al., 1999; Marriott et al., 1999).

### Thalidomide (THALOMID^®^) and First Generation of IMiDS (1996–1998; Figure 5)

**FIGURE 5:**
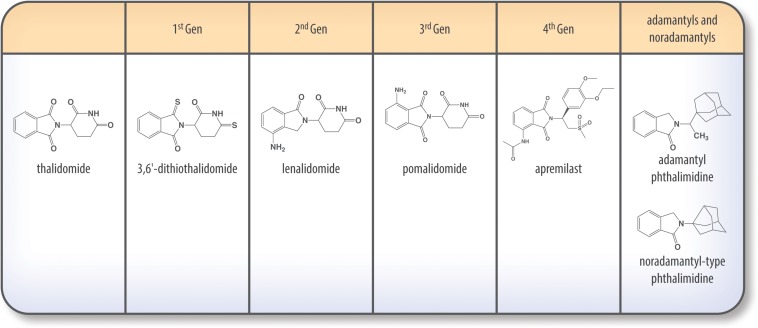
Chemical structures of thalidomide and its analogs.

Currently, thalidomide is used in the treatment of a broad number of inflammatory disorders such as Crohn’s disease, arthritis, ulcerative colitis, ENL, multiple myeloma, and dermal conditions involved with Behcet’s disease, as well as some types of cancers (Sheskin, 1965; Wettstein and Meagher, 1997; Haslett et al., 2005; FDA and CDER, 2014). Additionally, as already noted, due to its multipotent characteristics, thalidomide has been evaluated for repurposing across numerous neurological disorders.

In preclinical studies, both short-term and long-term thalidomide treatment of AD rodents led to decreases in overall disease pathology, neuroinflammation, and cognitive impairment (He et al., 2013; Kübra Elçioğlu et al., 2015). Thalidomide has also been shown to have anti-apoptotic effects in stroke models *in vitro* and *in vivo*. These effects are likely due to thalidomide’s induction of phosphorylated Akt and activation of the pro-growth PI3K/Akt pathway, which potentially counter the apoptotic effects associated with AD and stroke. Thalidomide’s protection against oxidative stress may also play a part in offsetting neuronal damage (Hyakkoku et al., 2009; Zhang et al., 2010; Farfán et al., 2015). Thalidomide has also shown promise in patients with blindness rising from TBM, with case studies of multiple patients being completely cured of blindness from a reduction of inflammation (Roberts et al., 2003; Schoeman et al., 2010). However, a pilot study of thalidomide treatment in children with TBM was terminated due to adverse effects (Schoeman, 2000).

To create the first generation of IMiDs, imide carbonyl groups of the thalidomide backbone were thionated in specific key positions. The resulting thiothalidomide analogs possess enhanced TNF-α inhibition, with di- and tri-thionated species demonstrating enhanced anti-TNF activity relative to standard thalidomide (Zhu et al., 2003). TNF-α lowering potency increased with the number of thions added to the glutarimide backbone, with trithiothalidomide decreasing TNF-α levels 30-fold more effectively than thalidomide (Greig et al., 2004; Tweedie et al., 2009).

3,6′-Dithiothalidomide (3,6′-DT), a compound developed and evaluated within our laboratory, demonstrated a decrease in microglial activation, TNF-α levels, and chronic inflammation in LPS-induced cell and rat models. In rats, treatment restored cognitive function and normalized plasticity-mediating gene expression in the hippocampus (Belarbi et al., 2012).

In mild TBI (mTBI) models, 3,6′-DT ameliorated all effects of injury in mice when treatment was administered from 1 to 12 h post-injury. These effects involved reversal of mTBI-induced decreases in cognitive function, increases in activated astrocytes, neuronal loss, increased TNF-α, and increased apoptosis in a mouse model of concussive injury (Baratz et al., 2011, 2015). These actions translated to a moderate to severe rat model of TBI in which 3,6′-DT significantly reduced contusion volume, neuronal degeneration, neuronal apoptosis and neurological deficits when administered within 5 h of injury, substantially lowering pro-inflammatory cytokines at the transcription and translation level, and suppressing neuronal markers of oxidative stress (Batsaikhan et al., 2019).

In AD mouse models of stereotaxic intracerebroventricular Aβ_1-42_, 3,6′-DT ameliorated Aβ-induced neuroinflammation and microglial activation, prevented neurodegeneration, and improved memory (Russo et al., 2012). In 3xTg (APP_Swe_/PS1_M146V_/tau_P301L_) AD mice, long term thalidomide or 3,6′-DT treatment stabilized TNF-α gene expression and protein levels to WT levels. However, thalidomide treatment failed to prevent cognitive decline in 3xTg mice’s working memory, whereas 3,6′-DT treatment successfully achieved this. Furthermore, only 3,6′-DT treatment increased the ratio of resting and activated microglia, which is associated with neuroprotection (Gabbita et al., 2012). 3,6′-DT treatment of 3xTg AD mice also resulted in lower hippocampal Aβ levels, tau deposits, and improved cognition (Gabbita et al., 2012; Tweedie et al., 2012).

In focal ischemic stroke mouse models, 3,6′-DT proved to be efficacious when administered 1–3 h post-stroke to reduce neuronal loss, inhibit TNF-α, IL-1β, iNOS, and apoptosis, whereas thalidomide showed no significant effect. Notably, TNFR KO mice were not rescued with 3,6′-DT post-injury, consistent with a critical role for the suppression of TNF-α production and TNF-α signaling in the therapeutic action of 3,6′-DT, and the value of potent TNF-α suppressing agents to restrain microglial activation and inflammation (Yoon et al., 2013). In separate studies, 3,6′-DT treatment of mice post-hypoxia or blast injury attenuated injury-related increases in TNF-α and changes in synaptic transmission (Wall et al., 2015; Wang et al., 2018), thereby, cross-validating the suppression of TNF-α generation and release to mitigate inflammation-mediated neuronal damage across animal models of CNS injury. Finally, in a recent study, both 3,6′DT and thalidomide were demonstrated to mitigate the severity of L-dopa-induced dyskinesia in nigrostriatal-lesioned rat models of PD, suppressing both microgliosis and elevated TNF-α in the striatum and substantia nigra, and restoring IL-10 anti-inflammatory cytokine levels (Boi et al., 2019).

### Second Generation – Lenalidomide (REVLIMID^®^) (FDA Approved 2006; Figure 5)

Lenalidomide, marketed as Revlimid by Celgene, is an analog of thalidomide with an amino group on the C4 position of the benzene ring system. Cellular studies have shown lenalidomide to activate T cells, suppress angiogenesis, and modulate inflammation by decreasing cytokines (Melchert and List, 2007). The drug induces M2 polarization of macrophages by upregulating IL-10 and activating STAT3, acting against inflammation to promote survival in MS models (Weng et al., 2018). It also downregulates inflammation-related miRNA in astrocyte cultures stimulated with LPS and MRP8 (Omran et al., 2013). Lenalidomide has also been reported to be 2,000-fold more potent than thalidomide in inhibiting TNF-α secretion from monocytes (Vallet et al., 2008). Lenalidomide treatment of ALS transgenic mice resulted in enhanced motor performance, decreased motor neuron death, and increased life span (Kiaei, 2006). Lenalidomide and, in particular, combination therapy with ROS scavenger Nanoceria were shown to ameliorate cognitive decline, CNS myelin loss, ventricular volume enlargement, neuroinflammation, and white matter damage in a MS model in mice (Eitan et al., 2015). Thalidomide and lenalidomide have also been shown to reduce microgliosis and NF-κB activation, motor deficits, and excitotoxicity and DA fiber loss in the striatum of PD mouse models (Palencia et al., 2015; Valera et al., 2015), supporting the potential use of IMiDs as a treatment for PD.

Lenalidomide was approved by the FDA in May 2006 for combination treatment with dexamethasone for relapsed or refractory multiple myeloma, and has undergone clinical trials for an increasing number of solid tumor malignancies, including prostate cancer, melanoma, and glioma (Aragon-Ching et al., 2007). It was also approved to treat myelodysplastic syndromes in 2005 (List, 2006; Melchert and List, 2007). The drug is currently in a phase 2 trial for relapsed CNS myeloma treatment (Rubenstein et al., 2018), and appears to have fewer adverse effects compared to thalidomide in the treatment of multiple myeloma. Notably, thalidomide’s often prominent side effects of constipation, peripheral neuropathy, and drowsiness, were not observed in lenalidomide clinical trials (Richardson et al., 2002), suggesting that these may not necessarily be drug-class dependent. Lenalidomide was determined to be safe in clinical trials for refractory melanoma, with T-cell stimulatory effects in cancer patients (Bartlett et al., 2004b). However, its association with increased risk for stroke was recently observed in multiple myeloma patients (de Celis et al., 2018). Although lenalidomide is not associated with the level of neurotoxicity that accompanies thalidomide (where peripheral neuropathy is reported in up to 83% of myeloma patients, Dalla Torre et al., 2016), with the longer use of lenalidomide reports of neurotoxicity – albeit milder than thalidomide and sometimes subclinical – are appearing in the literature (Dalla Torre et al., 2016). In contrast, venous thromboembolism has emerged as lenalidomide’s most common side effect (Bartlett et al., 2004a; Melchert and List, 2007), which may potentially be reduced when used in combination therapy with aspirin (Baz et al., 2005).

There is limited information in relation to the utility of lenalidomide in neurodegenerative disorders, but its evaluation in a PD α-synuclein mouse model demonstrated promising activity. It reduced motor behavioral deficits and mitigated DA fiber loss in the striatum. Microgliosis was decreased in both the striatum and hippocampus, and pro-inflammatory cytokines expression levels were decreased leading to a reduction in NF-κB activation (Valera et al., 2015).

### Third Generation – Pomalidomide (POMALYST^®^) (FDA Approved 2013; Figure 5)

Pomalidomide, marketed by Celgene under the name Actimid, is one of the most potent thalidomide derivatives to be discovered thus far. In a rat model of moderate to severe TBI, pomalidomide treatment ameliorated injury-induced neurodegeneration, apoptosis, cognitive decline, and proinflammatory cytokine upregulation when given up to 5 h post-injury. The same effects were observed in primary microglia and cortical cell cultures challenged with glutamate in a dose-dependent manner (Wang et al., 2016a). As shown in oxidative stress models, pomalidomide has neuroprotective and antioxidative effects mediated by Nrf2/SOD2/catalase pathways (Tsai et al., 2018). Finally, a recent study has demonstrated the activity of pomalidomide post-injury administration in a rat model of ischemic stroke, reducing the cerebral infarct volume and mitigating functional deficits (Tsai et al., 2019). A recent novel compound of interest, 3,6′-dithiopomalidomide (3,6′-DP), combines the advantages of thionation evident in 3,6′-DT on the thalidomide backbone into third generation pomalidomide. Preliminary studies demonstrate the potent action of 3,6′-DP in rodent TBI at one fifth of the efficacious dose of pomalidomide, and further results in other neuroinflammatory cellular and animal models are awaited with interest.

As noted earlier, recent studies have shown that IMiDs, excluding apremilast, bind to cereblon. Cereblon interacts with the DNA damage-binding protein-1 (DDB1), Cullin 4 (Cul4A or Cul4B), and regulator of Cullins 1 (RoC1) to create the functional E3 ubiquitin ligase complex. Within this ligase complex, cereblon acts as a substrate receptor to target select proteins for proteolysis via a ubiquitin-proteasome pathway (Shi and Chen, 2017). Cereblon appears to be one of many substrate receptor proteins that can be recruited into the E3 ubiquitin ligase complex to allow it to target different substrates. In this regard, IMiDs have been reported to form a molecular bridge between cereblon, within the E3 ubiquitin ligase complex, and its target protein to destine it for proteasome degradation. The glutarimide moiety of the IMiD binds cereblon, and the phthalimide moiety binds to the target proteins (Chamberlain et al., 2014; Fischer et al., 2014). Known targeted proteins, such as IKZF1/3, are predicted to possess a β-hairpin motif that comes into contact with the phthalimide structure (Petzold et al., 2016). Thalidomide, lenalidomide, or pomalidomide treatment hence leads to diminished levels of IKZF1/3, and likely other select proteins, which causes upregulation of IL-2 and activates T cells (Haslett et al., 2005; Gandhi et al., 2014). Pomalidomide appears to be the most potent with regards to T cell co-stimulation, but appears to have similar anti-angiogenic activity to that of lenalidomide (Bartlett et al., 2004b). Pomalidomide effectively modulates tumorigenesis and angiogenesis, making it a favorable drug for use in cancer treatment. It has been approved for use in multiple myeloma patients for whom lenalidomide and bortezomib have failed to prevent disease progression (Ríos-Tamayo et al., 2017).

### Fourth Generation – Apremilast (Otezla^®^) (FDA Approved 2014; Figure 5)

Apremilast was FDA approved to treat moderate to severe cases of plaque psoriasis and psoriatic arthritis in 2014 (Fala, 2015). Similar to other IMiDs, apremilast inhibits TNF-α protein synthesis. However, as noted previously, the drug acts via PDE4 inhibition, upregulating cAMP and thereby reducing proinflammatory cytokine production. Although apremilast is effective in treating peripheral inflammation, it has poor brain penetrance, as shown in *in vivo* pharmacokinetic studies using radioactivity to measure drug levels in the brain (Committee for Medicinal Products for Human Use [CHMP], 2014). LogP values of apremilast and other thalidomide analogs indicate apremilast has higher lipophilicity than other IMiDs, and its molecular size (460.5 Da) and general features are within the range of molecules that can cross the BBB (Table 2). However, *in vitro* confirmation of apremilast as a P-glycoprotein substrate, the low CNS MPO Score of apremilast in comparison to other IMiDs (Table 2), and its low brain/plasma concentration ratio of ≤0.1 in pharmacokinetic studies (Committee for Medicinal Products for Human Use [CHMP], 2014) support its lack of potential as an active drug for CNS disorders. There do not currently appear to be any literature reports of apremilast evaluation in animal models of neurodegeneration.

**TABLE 2:** Factors of drug pharmacokinetics at physiological conditions.

**Drug name**	**Molecular weight (g/mol)**	**cLogP**	**cLogD (pH7.4)**	**Number of Hydrogen bond donors**	**Number of Hydrogen bond acceptors**	**Topological polar surface area (Å^2^)**	**Strongest basic pK_a_ calculation**	**CNS MPO Score**
Thalidomide	258.2	0.02	0.02	1	4	83.55	11.59^∗^	4.8
3,6′-Dithiothalidomide	290.4	1.80	1.79	1	2	49.41	9.8^∗^	4.9
Lenalidomide	259.3	–0.71	–0.71	2	4	92.50	2.31	5.4
Pomalidomide	273.2	–0.16	–0.16	2	5	109.57	1.56	4.8
3,6′-Dithiopomalidomide	305.4	0.97	0.96	2	3	75.43	2.33	5.5
Apremilast	460.5	1.31	1.31	1	7	119.08	12.98^∗^	3.1
Adamantyl phthalimidine	295.4	3.86	3.86	0	1	20.31	–1.04	3.7
Noradamantyl phthalimidine	253.3	2.75	2.75	0	1	20.31	–0.85	4.7

*Molecular weight, cLogP/D value, number of Hydrogen bond donors/acceptors, topological polar surface area, and pK_*a*_ were generated using Chemicalize for several IMiDs, including a recent pomalidomide derivative of interest- 3,6′-dithiopomalidomide (3,6′-DP). IMiDs generally follow the Lipinski rule of five, which predicts the likelihood of a drug being delivered to its target in human physiological conditions based on its physical properties (Banks and Greig, 2019). A CNS MPO Score, which predicts drug BBB permeability, was calculated using parameters and functions described by Wager et al. (2010). Higher CNS MPO Score correlates with greater drug BBB permeability. ^∗^Indicates the calculated strongest acidic pK_a_ value used to calculate CNS MPO Score when the calculated strongest basic pK_a_ value was not provided in Chemicalize calculations. Calculated acidity values are not necessarily representative of the neutral parent compound and may instead reflect the pK_a_ of a corresponding protonated species.*

### Adamantyl and Noradamantyl Phthalimidines (Luo et al., 2018; Figure 5)

Adamantyl groups can be added to drugs to increase lipophilicity, drug stability, and plasma half-life (Liu J. et al., 2011). Adamantyl groups also strengthen molecule interactions with CNS targets, allowing development of more efficient drugs. For instance, iron chelator Desferrioxamine B (DFOB), which was seen to be effective in PD cell and animal models, was conjugated with adamantane and increased 66-fold in efficacy (Liddell et al., 2013). Likewise, many neuroprotective drugs such as memantine, one of the only drugs approved to treat AD, and amantadine, which is used to treat Parkinson disease, contain adamantyl groups (Rascol et al., 2011; Salomone et al., 2012). Another adamantyl derivative, *N*-adamantyl-4-methylthiazol-2-amine (KHG26693), suppresses oxidative damage caused by Aβ and LPS-induced increases in proinflammatory cytokines by suppressing NF-κB (Cho et al., 2015; Kim et al., 2017).

Our laboratory synthesized 15 novel IMiDs featuring adamantyl and noradamantyl phthalimidines. As noted earlier, the glutarimide and phthalimide moieties of thalidomide and analogs interact with cereblon and its targeted proteins, respectively, and modifications in these moieties could potentially influence the affinity and selection of targeted proteins (Yu et al., 2019). These 15 compounds have been shown to have nitrite and TNF-α lowering activity. The degree of nitration of adamantyl phthalimidines has also been shown to affect drug efficacy, with higher nitration yielding greater anti-nitrite activity (Luo et al., 2018). Interestingly, in a broader series of novel IMiDs likewise synthesized within our laboratory in which both small and substantial modifications were introduced into the glutarimide and phthalimide moieties, the resulting agents demonstrated a range of pharmacological actions (Beedie et al., 2016). Cohorts of compounds expressed anti-angiogenic properties, others anti-inflammatory properties and some exhibited both, thereby, allowing the separation of these and likely other actions. Compounds from these series are now being evaluated in animal models of neurodegeneration, and results are awaited with interest.

## Considerations Prior to IMiD Clinical Trials for Neurodegenerative Disease

Although thalidomide analogs are promising, there are still negative findings and drug adverse effects that must be addressed prior to its use for targeting inflammation in neurodegenerative disease.

First, thalidomide appears to be a pleiotropic drug, affecting multiple biological pathways upon entering the circulatory system. Although the multipotency of thalidomide gives the drug potential to treat a multitude of symptoms via many positive pathways, it also increases the risk for drug side effects. For instance, thalidomide has been very effective in healing orogenital ulceration via its anti-inflammatory effects while simultaneously inducing or exacerbating erythema nodosum via its T-cell costimulatory effects in Behcet syndrome patients (Hamuryudan et al., 1998). Thalidomide’s co-stimulation of T-cells has also been observed in a clinical trial of thalidomide in HIV patients (Corral et al., 1999; Vergara et al., 2017), pointing to the possibility of thalidomide inducing inflammation in immunocompromised individuals. The mechanisms underlying other adverse actions, such as the teratogenic effect, have yet to be fully characterized but are postulated to consist of a combination of thalidomide/metabolite interactions with the bodily environment, including thalidomide binding with cereblon, which yields angiogenesis inhibition (Vargesson, 2015). Additionally, recent research reveals that thalidomide interacts with cereblon to target and repress SALL4, a key transcription factor of the Spalt-like family with a critical role in embryonic limb development (Donovan et al., 2018; Matyskiela et al., 2018; Vargesson, 2019). Interestingly, nitric oxide has been shown to diminish thalidomide-induced teratogenicity by 80–94% (Siamwala et al., 2012), shedding light on the possibility of interventions that can be used to potentially mitigate adverse effects of IMiDs.

Another factor to consider is the multifaceted nature of neuroinflammation. Neuroinflammation sometimes promotes recovery from injury and is necessary at certain time points. For example, 50% of mTBI patients experience meningeal vascular injury from which they recover via actions of inflammatory myeloid cells. Subduing inflammation post-injury may potentially prevent positive effects of inflammation (Russo et al., 2018). As the inflammatory response is time-dependent and multi-faceted, IMiDs, which target both TNFR1 and TNFR2 activation, must be administered at timepoints corresponding to the detrimental effects of inflammation when treating various neurological diseases.

After the most efficacious thalidomide analogs are chosen for clinical studies following preclinical research, phase I trials to evaluate their safety and efficacy in humans with neurodegenerative diseases must be designed and conducted. As thalidomide analogs prevent and mitigate inflammation, clinical trials should be focused on AD patients that demonstrate elevated biomarkers associated with increased neuroinflammation.

Testing the efficacy of thalidomide analogs in conjunction with other drugs targeting different aspects of inflammation (i.e., RXR/PPARγ agonists) or neurodegeneration to enhance neuroprotective effects could also be taken into consideration. Furthermore, as enhancing oral bioavailability of the drug has been an issue due to low drug solubility, finding ways to enhance drug solubility while maintaining its oral delivery method is also critical. Delivering lower doses of thalidomide and analogs via solid dispersions is a potential alternative that can increase bioavailability (Barea et al., 2017), especially for patients with diminished drug absorption abilities.

## Summary

Repurposing drugs already on the market and modifying existing pharmacologic structures could yield therapeutic interventions capable of meeting pressing needs for neurological disease. Among drugs already on the market, thalidomide has shown great success despite its controversial history, yielding several analogs that have been approved for the treatment of diseases ranging from multiple myeloma to arthritis. Recent clinical studies of thalidomide have demonstrated that its preclinical promise in animal models of neurodegeneration does not translate to human disease consequent to dose-limiting adverse actions. Translational studies of second, third and fourth generation drugs have yet to be undertaken, and there is recent promising preclinical data on pomalidomide that, if cross-validated, may be supportive of human trials. Small, open label clinical trials of the anti-TNF-α antibody strategy involving perispinal injection suggest that targeting TNF-α is a reasonable approach to potentially treat a range of neurodegenerative disorders. Recent medicinal chemistry studies on the backbone of the classical glutarimide and phthalimide moieties that are common to thalidomide, lenalidomide, and pomalidomide indicate that we can separate pharmacological actions from one another. By altering the chemical structure of thalidomide and its analogs, we may be able to increase potency and bioavailability of IMiDs, and reduce their adverse action, possibly expanding their use to target neuroinflammation to ameliorate symptoms of neurological disease and even slow their progression.

Preclinical studies on TNF-α inhibition using thalidomide and IMiDs in models of diseases such as ALS, PD, AD, and TBI have shown promise, indicating the potential for the advancement of select members of this drug class from the bench to clinical trials and eventually, to the bedside of patients of neurological disease in need of treatment.

## Author Contributions

YJ wrote the manuscript and designed the figures. NG provided input and revised the manuscript. DT and MS provided input and direction in structuring of the manuscript. MS provided the chemical structures of the IMiDs.

## Conflict of Interest

NG and DT are named inventors on patents covering novel thalidomide analogs and have assigned all their rights to the National Institute on Aging, NIH. The remaining authors declare that the research was conducted in the absence of any commercial or financial relationships that could be construed as a potential conflict of interest.

## Abbreviations

A β: Beta-amyloid
Ach: acetylcholine
AD: Alzheimer’s disease
ALS: amyotrophic lateral sclerosis
APP: amyloid precursor protein
ARE: AU-rich element
BBB: blood–brain barrier
CCI: controlled cortical impact
COX-2: cyclooxygenase-2
ChEI: cholinesterase inhibitor
CNS: central nervous system
CSF: cerebrospinal fluid
DA: dopaminergic
DAMP: damage-associated molecular pattern
HD: Huntington’s disease
IFN: interferon
IL: interleukin
IMiD: immunomodulatory imide drug
iNOS: inducible nitric oxide synthase
LPS: lipopolysaccharide
LTD: long-term depression
LTP: long-term potentiation
MAPK: mitogen-activated protein kinases
MS: multiple sclerosis
mTBI: mild traumatic brain injury
NF- κ B: nuclear factor kappa-light-chain-enhancer of activated B cells
PAMP: pathogen-associated molecular pattern
PD: Parkinson’s disease
PNS: peripheral nervous system
PPAR γ: peroxisome proliferator-activated receptor gamma
pTau: phosphorylated tau
ROS: reactive oxygen species
TBI: traumatic brain injury
TBM: tuberculous meningitis
TGF- β: transforming growth factor beta
TLRs: toll-like receptors
TNF- α: tumor necrosis factor alpha
UTR: untranslated region
